# Supplementary material for: Splice-Junction-Based Mapping of Alternative Isoforms in the Human Proteome
Source: Cell Rep. Author manuscript; Available in PMC 2020 Jan 15. (PMC6961840; doi:10.1016/j.celrep.2019.11.026)
Supplement: 2 [file NIHMS1546469-supplement-2.zip › DF1/PXD006675/CardiacFibroblast/CardiacFibroblast_29_DAZAP1_LSSDVAGK.pdf]

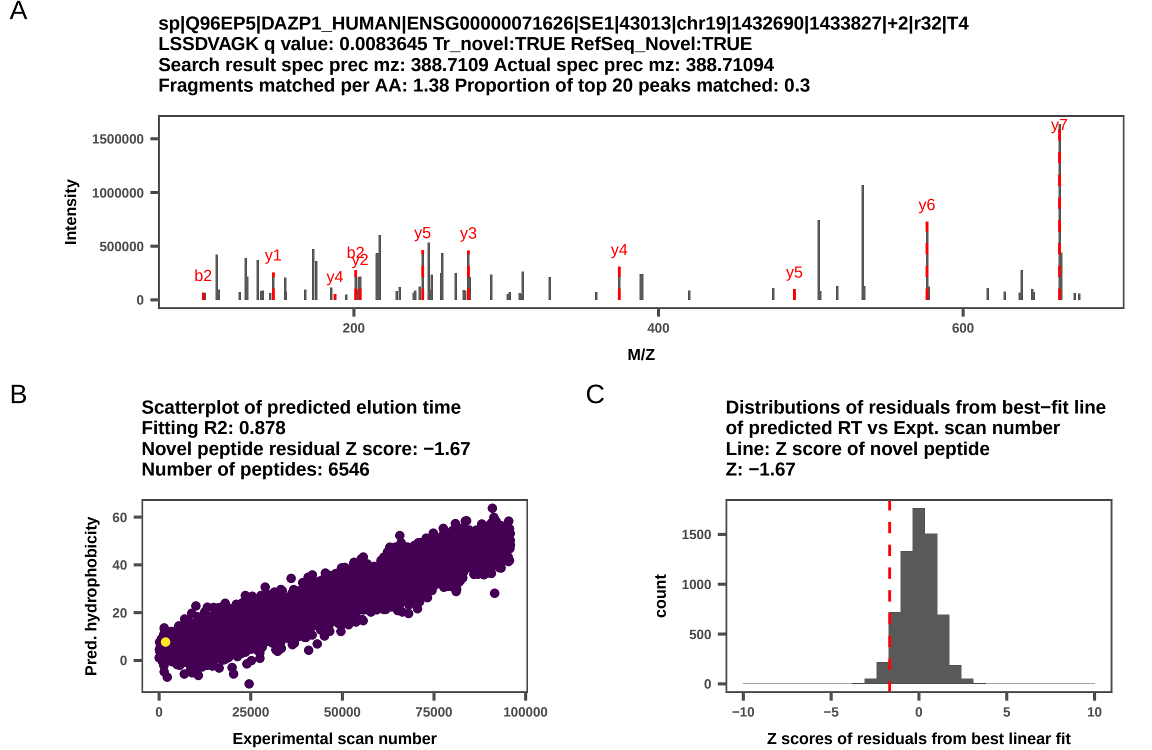
A
sp|Q96EP5|DAZP1_HUMAN|ENSG00000071626|SE1|43013|chr19|1432690|1433827|+2|r32|T4
LSSDVAGK q value: 0.0083645 Tr_novel:TRUE RefSeq_Novel:TRUE
Search result spec prec mz: 388.7109 Actual spec prec mz: 388.71094
Fragments matched per AA: 1.38 Proportion of top 20 peaks matched: 0.3
Intensity
M/Z
b2
y1
y4
b2
y2
y5
y3
y4
y5
y6
y7
B
Scatterplot of predicted elution time
Fitting R2: 0.878
Novel peptide residual Z score: −1.67
Number of peptides: 6546
Pred. hydrophobicity
Experimental scan number
C
Distributions of residuals from best−fit line
of predicted RT vs Expt. scan number
Line: Z score of novel peptide
Z: −1.67
count
Z scores of residuals from best linear fit
